# Supplementary material for: Motility-dependent processes in Toxoplasma gondii tachyzoites and bradyzoites: same same but different
Source: bioRxiv. 2024 Sep 28:2024.09.28.615543. Preprint. [Version 1] doi: 10.1101/2024.09.28.615543 (PMC11463423; doi:10.1101/2024.09.28.615543)
Supplement: 1 [file NIHPP2024.09.28.615543V1-supplement-1.pdf]

bioRxiv preprint doi: https://doi.org/10.1101/2024.09.28.615543; this version posted September 28, 2024. The copyright holder for this preprint (which was not certified by peer review) is the author/funder, who has granted bioRxiv a license to display the preprint in perpetuity. It is made available under aCC-BY 4.0 International license.

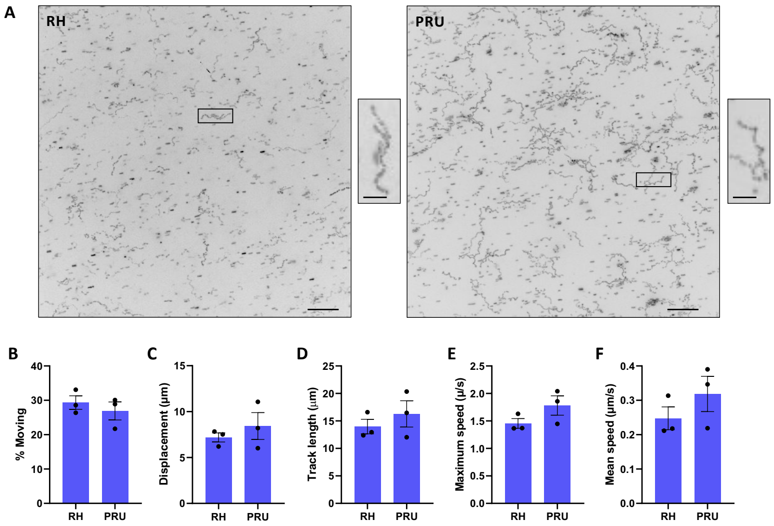

Supplementary figure 1. Comparison of RH and PRU tachyzoite motility.
(A) Representative maximum intensity projections of RH (left panel) and PRU (right panel) tachyzoites moving through Matrigel during 60 seconds of imaging; scale bar = 40μm. Insets (black boxes) are magnified, rotated and displayed to the right of each full field of view; scale bar 10μm. (B) Percentage of parasites moving > 2μm during 60 seconds of imaging; total number of parasites analyzed = 9064 (RH) and 7788 (PRU). (C-F) For all moving parasites, the following median trajectory parameters were quantified: C) displacement (distance from first to last point); D) track length; E) maximum speed achieved across the entire track; and F) mean speed. Each pair of motility parameters (RH vs PRU) was compared using an unpaired students t-test, and no significant difference in these parameters was identified between the strains. On the graphs, each data point represents one of three biological replicates, each consisting of 2 – 4 technical replicates. Bar height shows the mean and error bars show the s.e.m. of the biological replicates.

bioRxiv preprint doi: https://doi.org/10.1101/2024.09.28.615543; this version posted September 28, 2024. The copyright holder for this preprint (which was not certified by peer review) is the author/funder, who has granted bioRxiv a license to display the preprint in perpetuity. It is made available under aCC-BY 4.0 International license.

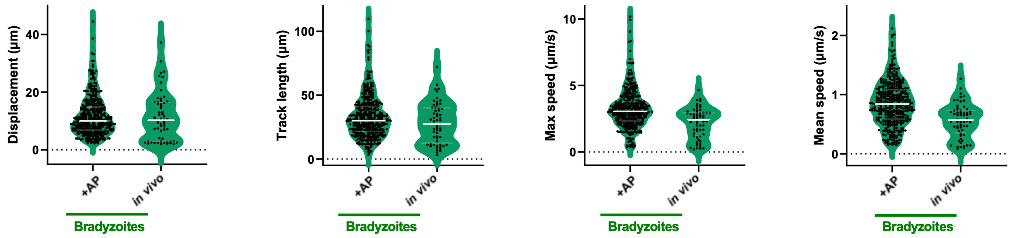

Supplementary figure 2. Comparison of the motility parameter distributions for *in vitro*- *vs. in vivo*-derived bradyzoites

(A) Displacement, (B) Track length, (C) Maximum speed and (D) Mean speed, for all motile parasites in the *in vitro* (plus acid pepsin digest) and *in vivo* populations, plotted as violin plots. The median of each distribution is shown as a horizontal white line. Using unpaired students t-tests; no significant differences were identified between the two populations for any of the four parameters when comparing the median, 5% or 95% values.

bioRxiv preprint doi: https://doi.org/10.1101/2024.09.28.615543; this version posted September 28, 2024. The copyright holder for this preprint (which was not certified by peer review) is the author/funder, who has granted bioRxiv a license to display the preprint in perpetuity. It is made available under aCC-BY 4.0 International license.

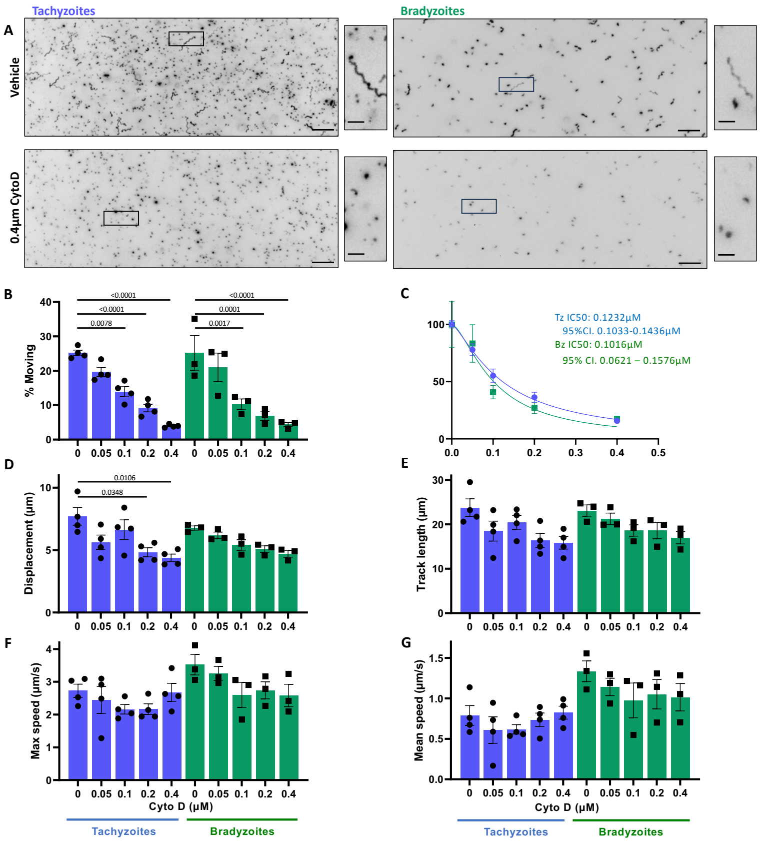

Supplementary figure 3. Comparison of tachyzoite and bradyzoite motility in the presence of Cytochalasin D.

(A) Representative maximum intensity projections of tachyzoites and bradyzoites moving through Matrigel during 60 seconds of imaging in the absence (top 2 panels) or presence (bottom 2 panels) of 0.4μM Cytochalasin D (CytoD); scale bar = 40μm. Insets (black boxes) are magnified, rotated and displayed to the right of each full field of view; scale bar 10μm. (B) Percentage of tachyzoites moving > 2.5μm and bradyzoites moving > 2.8μm during 60 seconds of imaging. (C) The IC50 for the % motility data shown in Panel A was calculated for both tachyzoites (blue) and bradyzoites (green); no significant difference was seen in their response to treatment, as indicated by the overlapping 95% confidence intervals (CI). (D-G) For all parasites that exceeded the 2.5/2.8μm displacement thresholds, the following median trajectory parameters were quantified: D) displacement; E) track length; F) maximum speed; and G) mean speed. On the graphs, each data point represents a biological replicate consisting of 2-3 technical replicates; bar height shows the mean and error bars show the s.e.m. of the biological replicates. The response of tachyzoites and bradyzoites at each concentration of CytoD was compared using unpaired students t-tests; no significant differences were identified between the two stages at any dose. The number of parasites analyzed in B-G was 4129, 2440, 1086, 906 (Tachyzoites 0, 0.05, 0.1, 0.2, 0.4μM CytoD respectively) and 2529, 1220, 1786, 1109, 568 (Bradyzoites 0, 0.05, 0.1, 0.2, 0.4μM CytoD respectively). The response of tachyzoites or bradyzoites to treatment with each concentration of compound was compared to the vehicle control (0) using an ordinary one-way ANOVA and Tukeys correction for multiple comparisons; only statistically significant differences ($p<0.05$) are shown.

bioRxiv preprint doi: https://doi.org/10.1101/2024.09.28.615543; this version posted September 28, 2024. The copyright holder for this preprint (which was not certified by peer review) is the author/funder, who has granted bioRxiv a license to display the preprint in perpetuity. It is made available under aCC-BY 4.0 International license.

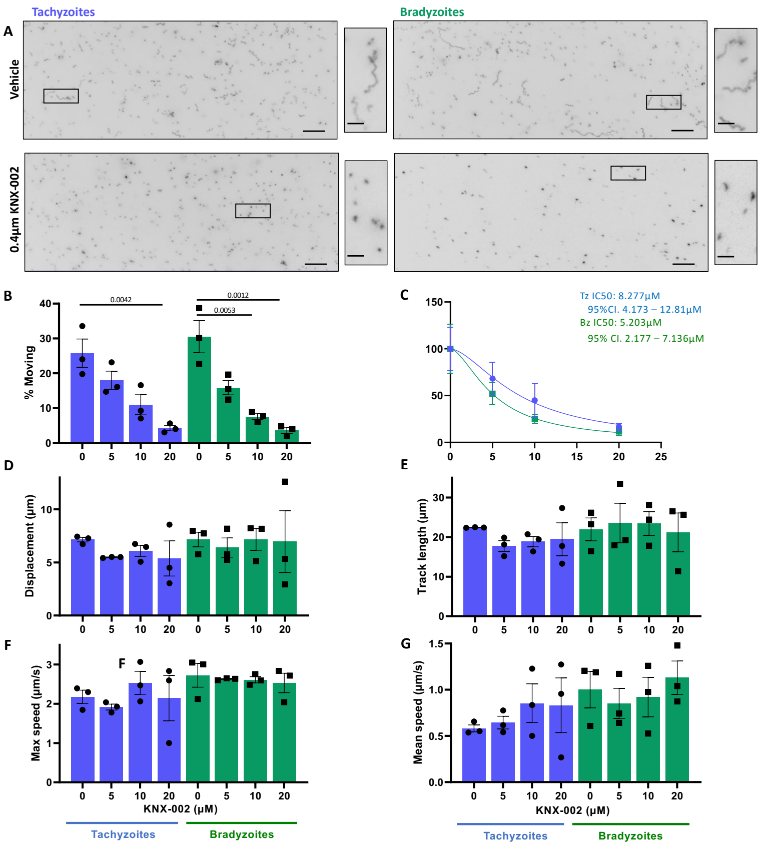

Supplementary figure 4. Comparison of tachyzoite and bradyzoite motility in the presence of KNX-002.

(A) Representative maximum intensity projections of tachyzoites and bradyzoites moving through Matrigel during 60 seconds of imaging in the absence (top 2 panels) or, presence (bottom 2 panels) of 20μM KNX-002; scale bar = 40μm. Insets (black boxes) are magnified, rotated and displayed to the right of each full field of view, scale bar 10μm. (B) Percentage of tachyzoites moving > 2.5μm and bradyzoites moving > 2.8μm during 60 seconds of imaging. (C) The IC50 for the % motility data shown in Panel A was calculated for both tachyzoites (blue) and bradyzoites (green); no significant difference was seen in their response to treatment, as indicated by the overlapping 95% confidence intervals (CI). (D-G) For all parasites that exceeded the 2.5/2.8 μm displacement thresholds, the following median trajectory parameters were quantified: D) displac8ement; E) track length; F) maximum speed; and G) mean speed. For each graph, each data point represents a biological replicate consisting of 2-3 technical replicates; bar height shows the mean and error bars show the s.e.m. of the biological replicates. The response of tachyzoites and bradyzoites at each concentration of KNX-002 was compared using unpaired students t-tests; no significant differences were identified between the two stages at any dose. The number of parasites analyzed in B-G was 2112, 1186, 1251, 715 (Tachyzoites 0, 5, 10, 20μM KNX-002 respectively), and 3108, 1199, 868, 600 (Bradyzoites 0, 5, 10, 20μM KNX-002 respectively). The response of tachyzoites or bradyzoites to treatment with each concentration of compound was compared to the vehicle control (0) with an ordinary one-way ANOVA and Tukeys correction for multiple comparisons; only statistically significant differences (p<0.05) are shown.

bioRxiv preprint doi: https://doi.org/10.1101/2024.09.28.615543; this version posted September 28, 2024. The copyright holder for this preprint (which was not certified by peer review) is the author/funder, who has granted bioRxiv a license to display the preprint in perpetuity. It is made available under aCC-BY 4.0 International license.

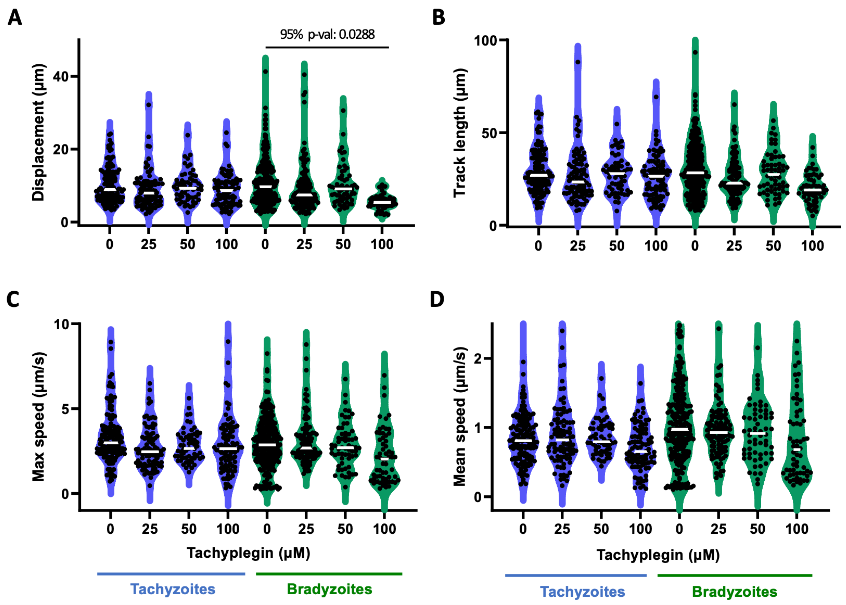

Supplementary figure 5. Comparison of the motility parameter distributions for tachyzoites and bradyzoites treated with tachyplegin.

Tachyzoites and bradyzoites were treated with increasing doses of tachyplegin. The motility parameters compared were (A) displacement, (B) track length (C) maximum speed achieved and (D) mean speed. The 5th and 95th percentile values for each stage and compound concentration were compared to the vehicle control (0) with an ordinary one-way ANOVA and Tukeys correction for multiple comparisons. The only statistically significant difference was a decrease in the 95th percentile of bradyzoite displacement, comparing 100μM tachyplegin to vehicle control.

bioRxiv preprint doi: https://doi.org/10.1101/2024.09.28.615543; this version posted September 28, 2024. The copyright holder for this preprint (which was not certified by peer review) is the author/funder, who has granted bioRxiv a license to display the preprint in perpetuity. It is made available under aCC-BY 4.0 International license.

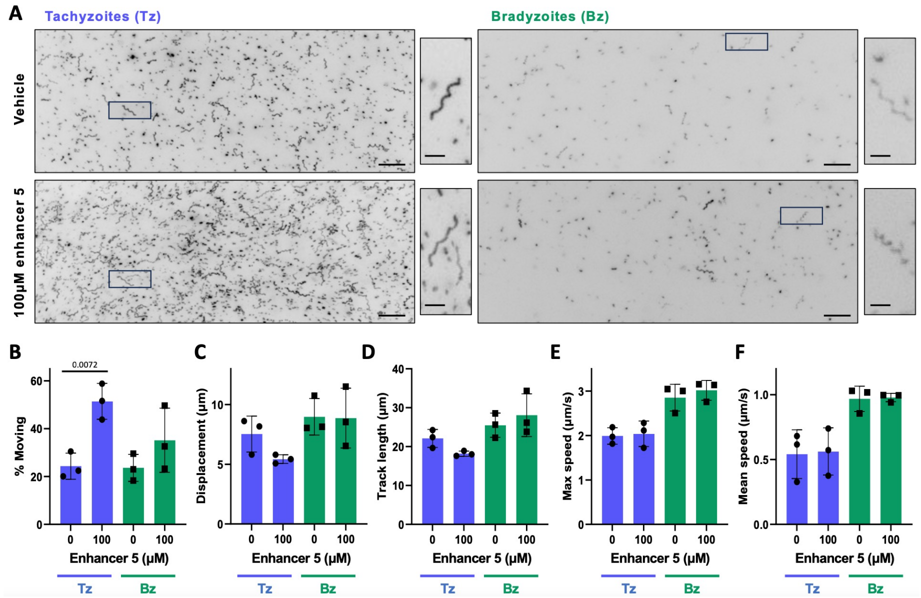

Supplementary figure 6. Comparison of tachyzoite and bradyzoite motility in the presence of enhancer 5.
(A) Representative maximum intensity projections of tachyzoites (Tz) and bradyzoites (Bz) moving through Matrigel during 60 seconds of imaging in the presence of 100μMol enhancer 5; scale bar = 40μm. Insets (black boxes) are magnified, rotated and displayed to the right of each full field of view; scale bar 10μm. (B) Percentage of tachyzoites moving > 2.5μm and bradyzoites moving > 2.8μm during 60 seconds of imaging. (C-F) For all parasites that exceeded the 2.5/2.8 μm displacement thresholds, the following median trajectory parameters were quantified: C) displacement; D) track length; E) maximum speed; and F) the mean speed. Each data point represents a biological replicate consisting of 2-4 technical replicates; top of the bars show the mean and error bars show the s.e.m. of the biological replicates. The number of parasites analyzed in B-F was 2359, 2810 (Tachyzoites 0, 100μM enhancer 5 respectively) and 1743, 2109 (Bradyzoites 0,100μM enhancer 5 respectively). The response of tachyzoites and bradyzoites to enhancer 5 was compared to vehicle only (0) using unpaired students t-tests; only significant differences (p<0.05) are shown.
